# Supplementary material for: 7,7″-Dimethoxyagastisflavone Inhibits Proinflammatory Cytokine Release and Inflammatory Cell Recruitment through Modulating ERα Signaling
Source: Biomedicines. 2021 Nov 26;9(12):1778. doi: 10.3390/biomedicines9121778 (PMC8698781; doi:10.3390/biomedicines9121778)
Supplement: Supplementary file 1 [file biomedicines-09-01778-s001.zip › biomedicines-1430789-supplementary.pdf]

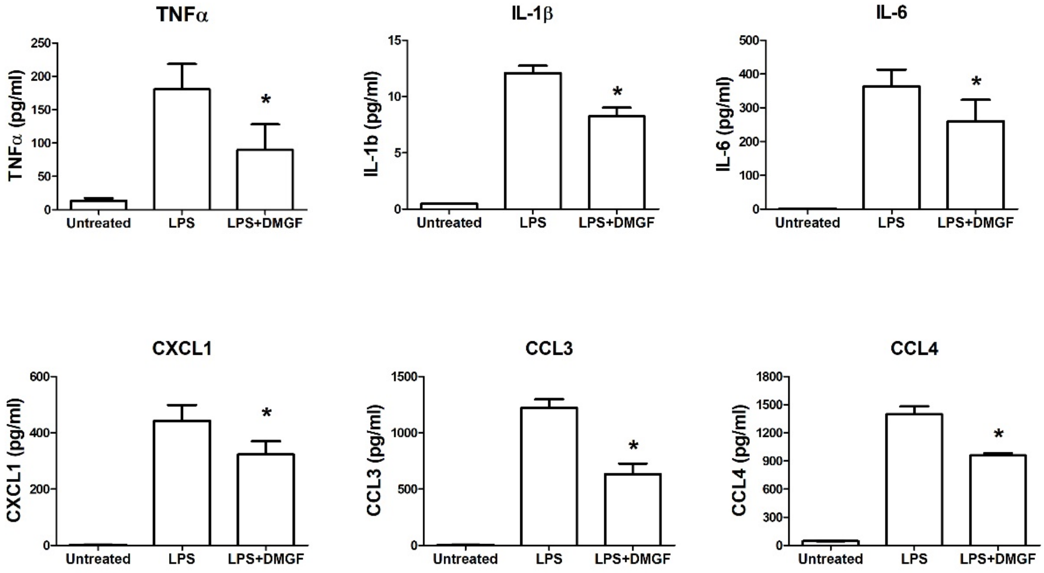

Figure S1. Effects of DMGF on cytokine and chemokine production. LPS-stimulated splenocytes were treated with 0.6 μg/ml DMGF for 48h. The concentration of TNFα, IL-1β, IL-6, CXCL1, CCL3, and CCL4 in the supernatants in each group was determined by a cytokine multiplex assay. *: $P<0.05$, compared to the LPS group.

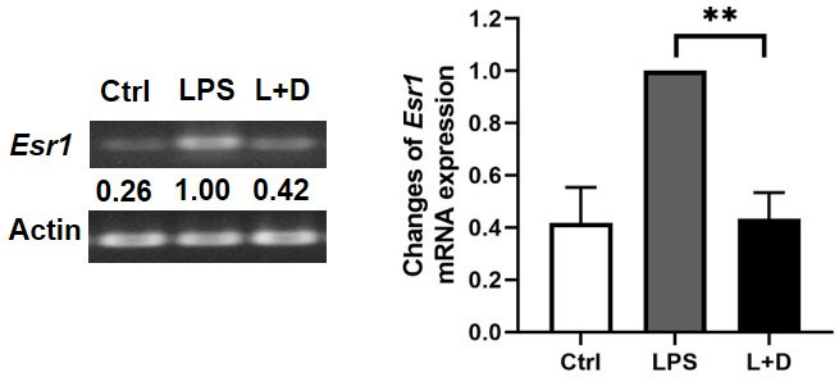

Figure S2. Effects of DMGF on mRNA expression of ERα. LPS-stimulated cells were treated with 0.6 μg/ml DMGF for 48h. ESR1 expression was determined by RT-PCR. The data of *Esr1* expression were normalized to the LPS group. **: *P*<0.01, compared to the LPS group.

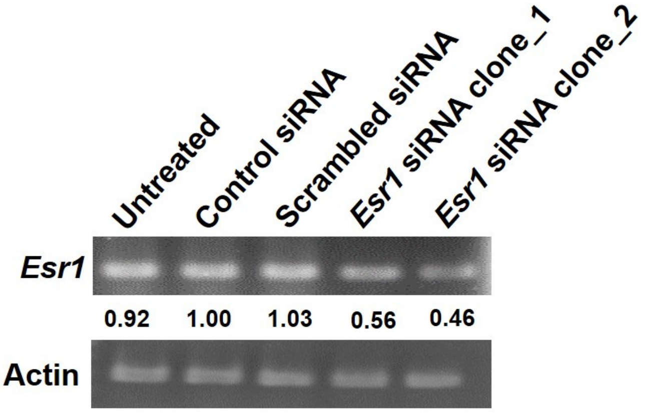

Figure S3. The knock-down efficiency of *Esr1* siRNA. After knockdown of *Esr1*-siRNA transfection to RAW264.7 cells, *Esr1* expression was confirmed by RT-PCR. The data of *Esr1* expression were normalized to the control siRNA. The experiments of TNFα, cell transmigration, integrin expression and and F-actin polymerization were performed in *Esr1* knockdown cells with *Esr1* siRNA clone_2 after DMGF treatment.

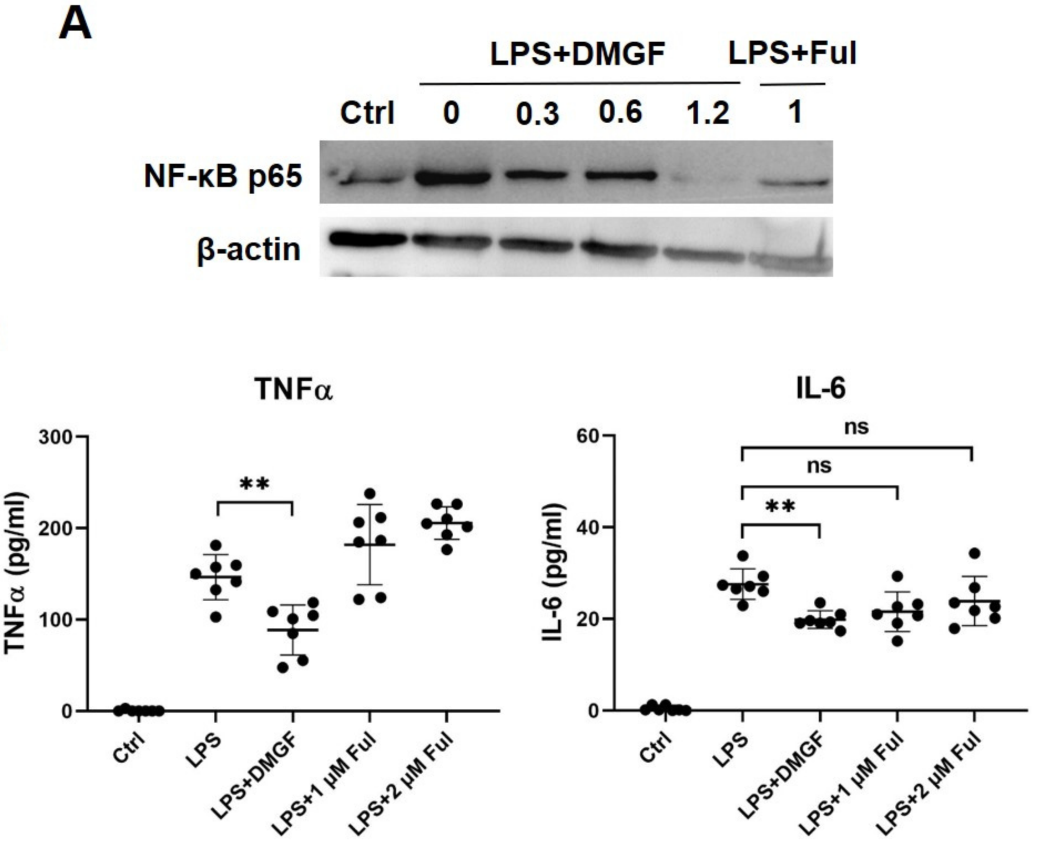

Figure S4. Effects of DMGF and fulvestrant on NF-κB signaling and proinflammatory cytokines release. (A) Total cellular NF-κB (p65) expression in LPS-stimulated splenocytes after DMGF treatment. LPS-stimulated splenocytes were treated with 0.6 μg/ml DMGF or 1 μM fulvestrant for 48h. Total cellular NF-κB (p65) expression was determined by western blot analysis. (B) The concentration of TNFα and IL-6 in the supernatants in each group was determined by ELISA. **: $P<0.01$, compared to the LPS group. ns: not significant.

Table S1. ESR1 transcription factor motifs

Promoter ID	Ensembl or RefSeq	ESR1 TF motifs
**Cytokines and chemokines**		
IL1B	NM_000576	-1240, -1125, -1026, -932, -551, -125
IL6	ENSG00000136244	-1937, -1214, -962, -808, -724, -558, -527, -120, 2
TNF	ENSG00000232810	-1403, -1348, -876
CXCL1	ENSG00000163739	-1888, -1355, -1278, -480, -476, -276, 87
CCL3	ENSG00000277632	-1597
CCL4	ENSG00000275302	-1309, -1263, -1096, -629, -624, -1
**Integrin**		
ITGAL	ENSG00000005844	-1932, -1353, -1002, -967, -814, -518, -74
ITGB2	ENSG00000160255	-1643, -1546, -1312, -1060, -899, -576, -493, -242, -119, -99
ITGA4	ENSG00000115232	-1412, 16, 87
**Actin polymerization molecules**		
CDC42	ENSG00000070831	-1848, -1397, -1014, -948, -927, -674, -431, -377, -353, -99, 51
PAK1	ENSG00000149269	-1485, -1487, -1538, -1918
WASL	ENSG00000106299	-1741, -1329, -1201, -951, -72, 56
RAC1	ENSG00000136238	-1475, -1143, -1070, -945, -753, -629, -430, -186
LIMK1	NM_002314	-1805, -1617, -1583, -1449, -1414, -1381, -1287, -1247, -1070, -1049, -855, -672, -648, 86
